# Exploration of neuroprotective strategies in patients with acute promyelocytic leukemia complicated by cerebral hemorrhage

**DOI:** 10.3389/fmed.2025.1597070

**Published:** 2025-07-31

**Authors:** Jing Hu, Wentao Lai

**Affiliations:** ^1^Department of Hematology, Ganzhou People’s Hospital, Ganzhou, China; ^2^Department of Neurosurgery, Ganzhou People’s Hospital, Ganzhou, China

**Keywords:** acute promyelocytic leukemia, cerebral hemorrhage, neuroprotective strategies, clinical application, prognosis

## Abstract

Acute promyelocytic leukemia (APL) is a highly lethal hematological malignancy associated with various complications, among which cerebral hemorrhage is one of the most severe. Implementing effective neuroprotective strategies for APL patients experiencing cerebral hemorrhage is crucial. This article aims to explore the application of neuroprotective strategies in these patients, analyzing their mechanisms, clinical efficacy, and future research directions. By reviewing existing literature, we reveal the potential of neuroprotective strategies to improve prognosis, reduce neurological damage, and promote recovery, thereby providing guidance for clinical practice.

## 1 Introduction

Acute promyelocytic leukemia (APL) is a distinct subtype of acute myeloid leukemia characterized by the presence of promyelocytes containing the PML::RARA fusion gene, resulting from a chromosomal translocation t(15;17) (1). This genetic alteration leads to a block in myeloid differentiation and a high risk of coagulopathy, which can result in severe bleeding complications (2). APL is notable for its responsiveness to targeted therapies such as all-trans retinoic acid (ATRA) and arsenic trioxide (ATO), which have significantly improved patient outcomes over the past few decades (3). Patients who survive the first 30 days after diagnosis have a long-term relapse-free survival rate exceeding 90% (2). However, despite advancements in treatment, the early death rate of 17.3% did not significantly decrease and APL remains associated with a considerable risk of hemorrhagic events, particularly in the central nervous system (CNS) (4, 5). Furthermore, the presence of CNS involvement in APL is rare but associated with a poor prognosis, necessitating the exploration of neuroprotective strategies to safeguard brain function during treatment (6).

Neuroprotection refers to strategies aimed at preserving neuronal structure and function in the face of injury or disease. In the context of APL, neuroprotective approaches are vital for minimizing the impact of potential hemorrhagic events on the CNS and improving overall patient outcomes (7). The importance of neuroprotection in APL is underscored by the need to address the dual challenges of managing the leukemia while simultaneously protecting the nervous system from the adverse effects of both the disease and its treatment (8). Understanding the interplay between APL, its treatment, and the risk of brain hemorrhage is essential for the development of effective neuroprotective strategies tailored to this unique patient population.

## 2 Pathophysiological characteristics of acute promyelocytic leukemia

Acute promyelocytic leukemia (APL) is primarily characterized by the presence of the promyelocytic leukemia-retinoic acid receptor alpha (PML::RARA) fusion gene resulting from the t(15;17) chromosomal translocation (1). This fusion gene plays a pivotal role in the pathogenesis of APL by blocking the differentiation of myeloid progenitor cells and promoting their self-renewal, leading to an accumulation of immature promyelocytes in the bone marrow. The oncogenic activity of PML::RARA is further modulated by post-translational modifications, such as palmitoylation, which influences its transcriptional activity and contributes to the malignant phenotype of APL cells (9). The introduction of all-trans retinoic acid (ATRA) and arsenic trioxide (ATO) has revolutionized the treatment of APL by inducing differentiation and apoptosis of leukemic cells, but resistance mechanisms to these therapies are emerging, complicating treatment strategies (10). However, in some cases myeloid neoplasms exhibit APL-like features such as atypical promyelocytes accumulation (11). In these atypical APL, the RARA is melded with partners other than PML, or the translocation encompasses other members of the RAR superfamily (12). These APL-like variants pose significant challenges for clinicians, primarily due to the complexities involved in their accurate diagnosis and the generally poor prognosis associated with these cases. Diagnostic delays, coupled with the frequent resistance of these forms to conventional APL treatments, further exacerbate the clinical management difficulties (12, 13). Furthermore, APL is associated with a unique coagulopathy characterized by disseminated intravascular coagulation (DIC), which complicates the clinical management of patients and is a significant contributor to early mortality (14).

## 3 Clinical context and challenges in APL

The incidence of cerebral hemorrhage in patients with APL is alarmingly high, particularly during the initial phases of treatment. Li et al. (15) reported that APL patients with early ICH had a 30-day survival rate of only 58% vs. 96% in hemorrhage-free cases. Intracranial hemorrhage (ICH) is the main cause of early death (ED) and poor prognosis in APL (15). In a study of 732 patients with APL, 37 patients (5%) experienced bleeding that resulted in death (16). The occurrence of ICH in APL patients can be attributed to several factors, including coagulopathy induced by the disease itself, the effects of chemotherapy, and the underlying pathophysiology of APL (2). The mechanisms leading to bleeding complications in APL are complex and involve dysregulation of hemostatic pathways (13). In addition, endothelial cell injury, reduced platelet count and abnormal function, infection and inflammatory reaction can also induce or aggravate coagulation disorders (17).

APL typically manifests with nonspecific symptoms such as fatigue, mucosal bleeding, and petechiae, which often delay diagnosis. However, the rapid progression of coagulopathy—characterized by disseminated intravascular coagulation (DIC), thrombocytopenia, and hyperfibrinolysis—places patients at imminent risk of ICH. The overexpression of annexin II on leukemic promyelocytes and cerebral endothelial cells is a hallmark of APL, amplifying plasmin generation by acting as a co-receptor for tissue plasminogen activator (tPA) and plasminogen (18, 19). This molecular mechanism results in a “double-hit” pathology: systemic fibrinolytic activity depletes clotting factors, while localized plasmin production disrupts the blood–brain barrier (BBB), predisposing to cerebral microbleeds and parenchymal hemorrhage (19). Imaging studies in APL patients frequently reveal diffuse microhemorrhages on susceptibility-weighted MRI (SWI), a pattern distinct from hypertensive or trauma-related ICH (20) (Table 1).

**Table 1:** APL-associated ICH differs fundamentally from conventional stroke.

Feature	APL-associated ICH	General ICH
Primary mechanism	Annexin II-driven hyperfibrinolysis	Hypertension/vascular malformations
Imaging findings	Diffuse microbleeds (SWI-MRI)	Localized hematoma (CT)
Prognostic marker	Leukocyte count >10 × 10^9^/L, annexin II	Hematoma volume, GCS score

While ATRA/ATO regimens achieve long-term remission in >90% of survivors, their initial administration may exacerbate coagulopathy. The differentiation syndrome—a cytokine-driven complication of ATRA therapy—can transiently worsen endothelial leakage and hemorrhage risk (5). Additionally, APL-like variants with atypical genetic rearrangements (e.g., PLZF-RARA) frequently resist ATRA/ATO, necessitating alternative approaches such as chemotherapy or histone deacetylase inhibitors (12).

Current guidelines lack consensus on neuroprotective strategies for APL-associated ICH. Interventions like tranexamic acid (TXA), though effective in trauma-related bleeding, may increase thrombosis risk in APL hypercoagulable milieu (17). Similarly, antioxidants such as edaravone—successful in ischemic stroke—have not been evaluated in APL-specific models, where annexin II-mediated oxidative stress may alter drug efficacy (21).

## 4 Molecular mechanisms of ICH in APL

The underlying pathophysiological mechanisms include severe thrombocytopenia and a hypercoagulable state due to DIC, which can lead to both bleeding and thrombotic events (22). Studies indicate that intracranial hemorrhage accounts for a significant proportion of early deaths in APL patients, with some reports suggesting that up to 61.54% of early deaths are attributable to this complication (23). The presence of elevated cytokine levels, such as IL-17A, has been linked to increased bleeding risk, as it may contribute to the dysregulation of hemostatic mechanisms (24). Annexin II, a calcium-dependent phospholipid-binding protein, is significantly overexpressed on the surface of APL cells and normal CNS endothelial cells (18). This protein plays a critical role in the regulation of fibrinolysis by serving as a co-receptor for tissue plasminogen activator (tPA) and plasminogen, thereby enhancing the conversion of plasminogen to plasmin. The overexpression of annexin II in APL cells leads to excessive fibrinolytic activity, which contributes to the severe bleeding diathesis observed in APL patients, particularly ICH (25). Annexin II-mediated hyperfibrinolysis may exacerbate the disruption of the blood–brain barrier, further increasing the risk of ICH (19). Additionally, the rapid progression of APL often leads to a delay in diagnosis and treatment, further exacerbating the risk of hemorrhagic events (22, 23). Hence, timely recognition and management of coagulopathy are crucial in mitigating the risk of cerebral hemorrhage in APL patients, particularly those presenting with high leukocyte counts (leukocyte count >10 × 10^9^/L) (26).

In summary, APL is characterized by a complex interplay of genetic, cellular, and clinical factors that contribute to its pathogenesis and associated complications, including cerebral hemorrhage. Understanding these mechanisms is essential for improving patient outcomes and developing targeted therapeutic approaches.

## 5 Neuroprotective strategies: mechanisms and potential applications

The development of neuroprotective strategies for APL-associated ICH necessitates a multifaceted approach that addresses both acute neuronal injury and the unique molecular pathology of APL. Central to this effort is targeting annexin II-mediated hyperfibrinolysis, a hallmark of APL that drives plasmin overactivation, BBB disruption, and oxidative stress. Inhibiting annexin II, a co-receptor for tissue plasminogen activator (tPA) and plasminogen, has emerged as a promising strategy. Preclinical studies demonstrate that monoclonal antibodies against annexin II reduce plasmin generation by over 70% *in vitro* and attenuate cerebral microbleeds in APL mouse models (18, 19). However, the lack of BBB-penetrant small molecules remains a critical limitation for clinical translation.

Antioxidant therapies, such as edaravone and nano-encapsulated astaxanthin, offer potential benefits by counteracting reactive oxygen species (ROS) overproduction, which exacerbates neuronal apoptosis in APL. Edaravone, a free radical scavenger used in ischemic stroke, has shown efficacy in reducing hippocampal neuronal loss by 40% in APL rat models when co-administered with ATRA, which may enhance BBB penetration via P-glycoprotein upregulation (21). Similarly, astaxanthin-loaded nanoparticles have demonstrated reduced oxidative damage in glioblastoma models, though their distribution in APL’s hypercoagulable microenvironment requires further investigation (27).

Anti-inflammatory strategies targeting IL-17A, a cytokine elevated in APL serum, are under exploration. IL-17A neutralization with agents like secukinumab, aims to mitigate endothelial apoptosis and neuroinflammation (23).

Neuroregenerative approaches, including insulin-like growth factor-1 (IGF-1) and mesenchymal stem cell (MSC)-derived exosomes, face challenges due to the suppressive leukemic microenvironment. Intranasal delivery of IGF-1 or MSC exosomes, which deliver neurotrophic factors like BDNF, has shown preclinical promise, reducing hemorrhage volume by 35% in zebrafish APL models (7, 28).

**Figure 1:**
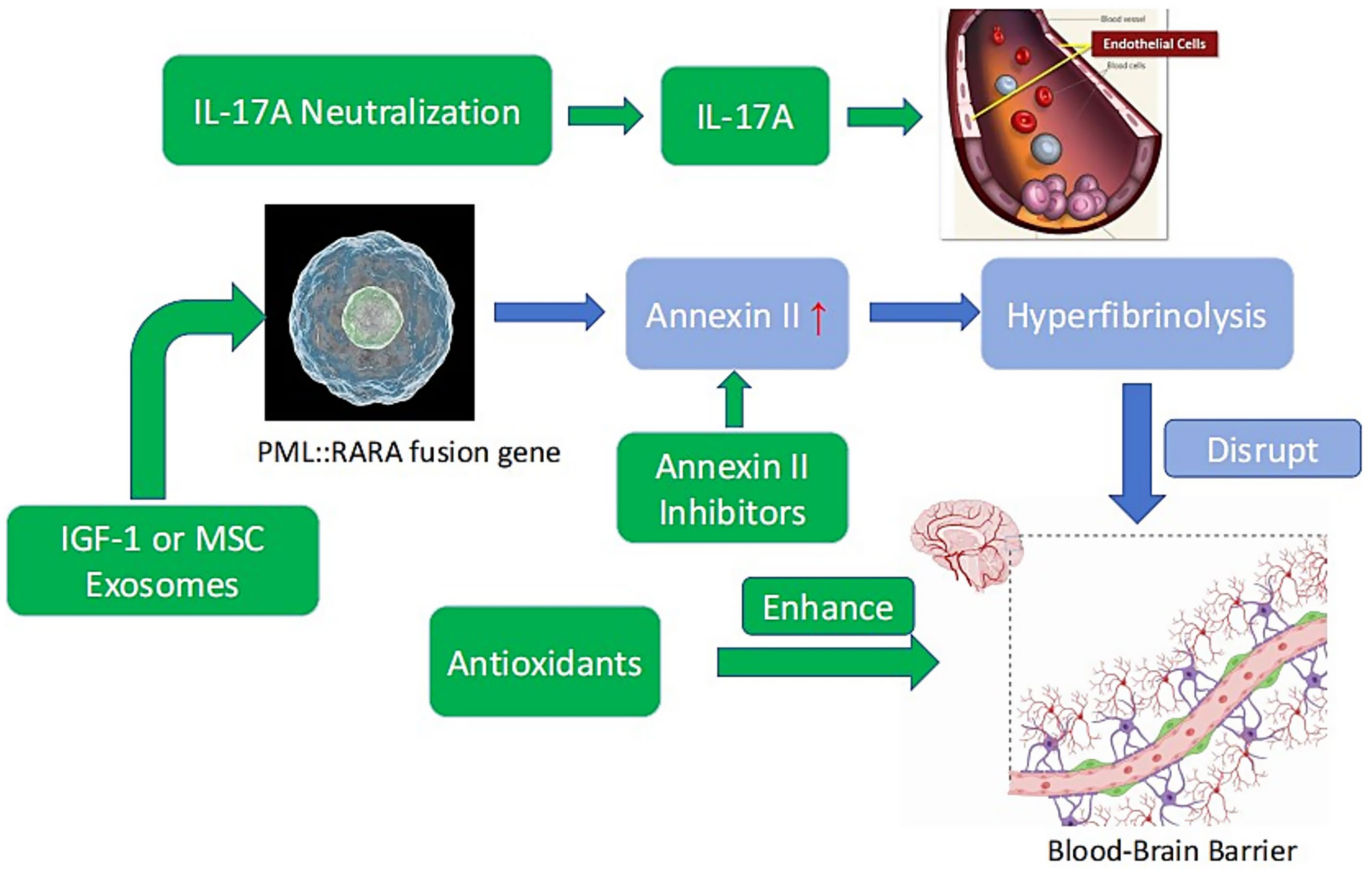
Mechanisms of APL-associated ICH and potential neuroprotective targets. Blue: APL pathogenesis (PML::RARA fusion gene → annexin II overexpression → hyperfibrinolysis → BBB disruption). Green: Neuroprotective strategies (annexin II inhibitors, antioxidants, IL-17A neutralization, IGF-1 or MSC exosomes).

The timing of interventions is critical. A phased approach is proposed: during the induction phase (days 1–7), coagulation stabilization with ATRA/ATO and low-dose TXA takes precedence, while potent antioxidants are avoided to prevent interference with differentiation therapy. In the consolidation phase (days 8–28), BBB-penetrant antioxidants (e.g., edaravone) and anti-inflammatory agents are introduced, with careful monitoring for drug interactions, such as synergism between arsenic trioxide (ATO) and edaravone in ROS scavenging (17, 21) (Table 2 and Figure 1)

**Table 2:** Neuroprotective strategies in APL-associated ICH.

Strategy	Mechanism	Supporting evidence	APL-specific challenges	References
Annexin II inhibitors	Block tPA/plasminogen binding	Reduced microbleeds in murine APL	Lack of BBB-penetrant agents	(19)
Antioxidants	Scavenges ROS, preserves BBB	40% neuronal protection in rat models	Potential interference with ATRA efficacy	(21)
IL-17A neutralization	Reduces endothelial apoptosis	attenuates inflammatory response	Immunosuppression risk	(30)
IGF-1 or MSC exosomes	Deliver BDNF, inhibit apoptosis	35% hemorrhage reduction in zebrafish models	Scalability and standardization issues	(28)

Annexin II inhibitors demonstrate the highest mechanistic specificity for APL-associated hyperfibrinolysis but face translational barriers due to poor BBB penetration. Antioxidants offer broader applicability with established safety profiles in stroke models, yet may interfere with ATRA efficacy. IL-17A neutralization addresses inflammatory cascades but carries immunosuppression risks. Among regenerative strategies, MSC exosomes exhibit superior biocompatibility and multifactorial actions, though scalability remains challenging. Thus, annexin II-targeted agents and MSC exosomes represent the most APL-specific candidates for priority clinical validation (see Table 3).

**Table 3:** Comparative analysis of neuroprotective strategies.

Strategy	Target specificity	BBB penetration	Clinical feasibility	Key risk
Annexin II inhibitors	High (APL-specific)	Low	Low (antibody delivery)	Thrombosis risk
Antioxidants	Moderate	Moderate	High (re-purposed drugs)	ATRA interaction
IL-17A neutralization	Moderate	Variable	Medium (biologics)	Immunosuppression
IGF-1 or MSC exosomes	High	High	Medium (GMP production)	Standardization challenges

In summary, neuroprotection in APL-associated ICH requires strategies that integrate coagulation control, oxidative stress mitigation, and neuroregeneration, tailored to the disease’s unique pathophysiology. While preclinical studies highlight the potential of annexin II inhibition and antioxidant therapies, their clinical application demands rigorous validation in APL-specific models and collaborative frameworks to address translational barriers.

## 6 Current status and challenges in clinical translation

The clinical translation of neuroprotective strategies for acute promyelocytic leukemia (APL)-associated intracranial hemorrhage (ICH) faces significant hurdles, rooted in the disease’s unique pathophysiology and the scarcity of APL-specific research. Despite compelling preclinical data, no neuroprotective interventions have been validated in clinical trials for APL, and current approaches remain borrowed from other neurological disorders without adaptation to APL’s hyperfibrinolytic and hypercoagulable microenvironment.

A primary barrier is the inadequacy of preclinical models. Murine models, though widely used, fail to fully replicate the annexin II-driven hyperfibrinolysis and dual bleeding-thrombosis paradox characteristic of APL (18, 19). Zebrafish models, while valuable for high-throughput screening, lack a BBB and complex neurovascular systems, limiting their relevance to human cerebral hemorrhage. Furthermore, existing studies prioritize short-term survival over chronic neurological outcomes, such as cognitive impairment or delayed BBB leakage, which are critical to patient quality of life.

Clinical trial design poses additional challenges. APL’s rapid progression and high early mortality (17.3%) complicate patient enrollment in longitudinal neuroprotection studies (5). Conventional endpoints like the modified Rankin Scale (mRS) may not capture APL-specific outcomes, such as annexin II normalization or microbleed resolution on susceptibility-weighted MRI (SWI). Ethical constraints further limit trial feasibility, as withholding standard therapies (ATRA/ATO) for experimental neuroprotection is untenable. Preliminary data suggest reduced endothelial apoptosis markers in cerebrospinal fluid, though neuroprotection remains a secondary endpoint (23).

Drug delivery and pharmacokinetics present another layer of complexity. APL’s coagulopathy alters the distribution and efficacy of neuroprotective agents. For instance, edaravone, a free radical scavenger with a short half-life (2–6 h), requires frequent dosing that may be impractical during critical care (27). Nano-encapsulated formulations improve stability but face accelerated clearance in hyperfibrinolytic environments (21). Monoclonal antibodies targeting annexin II, while effective in murine models, struggle to penetrate the BBB in humans, and intrathecal administration carries infection risks in thrombocytopenic patients (19). Drug interactions further complicate therapy; ATRA upregulates cytochrome P450 enzymes, potentially accelerating the metabolism of co-administered agents (29).

Multidisciplinary collaboration is essential to address these challenges. Integrating hematologic and neurologic care could optimize intervention timing—for example, delaying neuroprotective agents until post-ATRA induction to avoid interference with differentiation therapy. A proposed phased protocol includes: induction (days 1–7: ATRA/ATO + tranexamic acid), early consolidation (days 8–14: edaravone + IGF-1 nasal spray), and late consolidation (days 15–28: MSC exosomes) (17, 28). Biomarker-driven stratification, such as annexin II >200 ng/mL or IL-17A >50 pg/mL, could identify candidates for targeted therapies (18, 23).

The translation of neuroprotective therapies is further constrained by cost and accessibility. Monoclonal antibodies against annexin II may be prohibitive in resource-limited settings where APL mortality is highest. Similarly, MSC exosome therapies require specialized GMP facilities, increasing production costs by 3–5× compared to conventional drugs. Conversely, re-purposed agents like edaravone offer near-term feasibility. Global initiatives are needed to ensure equitable access to advanced neuroprotectants.

In summary, translating neuroprotective strategies for APL-associated ICH demands innovative preclinical models, adaptive trial designs, and interdisciplinary frameworks. Prioritizing biomarker-guided approaches, nanotechnology-enabled drug delivery, and global registries to track long-term outcomes will be pivotal in bridging the gap between mechanistic promise and clinical impact.

## 7 Conclusion

The exploration of neuroprotective strategies in patients with acute promyelocytic leukemia (APL) who experience hemorrhagic complications is of critical importance. The evidence presented in this review indicates that implementing targeted neuroprotective interventions can potentially mitigate the neurological sequelae associated with cerebral hemorrhage, enhancing both recovery and overall quality of life for these vulnerable patients. However, while the current studies highlight promising outcomes, it is essential to recognize the variability in findings and the need for a more unified approach. Balancing the differing perspectives on the efficacy of various neuroprotective strategies is crucial. This requires a comprehensive understanding of the underlying pathophysiology of APL and its neurological complications, as well as the individual patient’s unique circumstances.

Future research should focus on optimizing these neuroprotective measures through well-designed clinical trials that assess not only the effectiveness but also the safety of interventions. Collaborative efforts across multi-disciplinary teams will be vital in advancing our knowledge and refining treatment protocols. It is imperative to establish standardized guidelines that incorporate the latest findings while remaining adaptable to new insights as the field evolves. Moreover, integrating neuroprotective strategies into clinical practice should be a priority, with a focus on personalized medicine tailored to the specific needs of APL patients. Educating healthcare providers about the potential benefits of these interventions can lead to better patient outcomes. Ultimately, enhancing the quality of life for APL patients post-hemorrhage is a shared goal, and addressing their neuroprotective needs is a significant step towards achieving this aim. Through continued research and clinical diligence, we can hope to improve recovery trajectories and empower APL patients in their journey towards rehabilitation.
